# High-Strength GO/PA66 Nanocomposite Fibers via In Situ Precipitation and Polymerization

**DOI:** 10.3390/polym13111688

**Published:** 2021-05-22

**Authors:** Ao Gu, Jian Wu, Liming Shen, Xiaoyan Zhang, Ningzhong Bao

**Affiliations:** State Key Laboratory of Materials-Oriented Chemical Engineering, College of Chemical Engineering, Nanjing Tech University, Nanjing 210009, China; 201861104102@njtech.edu.cn (A.G.); jian.wu@njtech.edu.cn (J.W.); xzhang@njtech.edu.cn (X.Z.)

**Keywords:** Polyamide 66, GO, in situ precipitation, dispersion, mechanical properties

## Abstract

The uniform dispersion of graphene oxide (GO) and strong interfacial bonding are the key factors in achieving the high mechanical strength of GO/polymer composites. It is still challenging to prepare GO/PA66 composites with uniform GO dispersion by the in situ polymerization method. In this paper, we prepare GO/PA66 salt nanocomposite by in situ precipitating PA66 salt with GO in ethanol. The GO/PA66 nanocomposite fibers are then fabricated using the as-prepared GO/PA66 salt by in situ polymerizing and melt spinning. By tuning the GO content, the tensile strength and Young’s modulus of the GO/PA66 fibers are increased from 265 ± 18 to 710 ± 14 MPa (containing 0.3 wt% GO) and from 1.1 ± 0.08 to 3.8 ± 0.19 GPa (containing 0.5 wt% GO), respectively. The remarkable improvements are attributed to the uniform dispersion of GO in the GO/PA66 salt nanocomposite via ionic bonding and hydrogen bonding in the in situ precipitation process, and the covalent interfacial bonding between the GO and PA66 during the in situ polymerization process. This work sheds light on the easy fabrication of high-performance PA66-based nanocomposites.

## 1. Introduction

Polyamide 66 (PA66) is a very important thermoplastic polymer material and has been widely used in engineering fields with the advantages of high-strength, good stability, light weight, and relatively low cost [1,2,3]. However, with the rapid development of science and technology, the original PA66 can no longer meet the requirements of contemporary applications, especially in the fields that require high mechanical properties [4,5,6,7,8]. In order to meet the demands of new applications, high-performance PA66 composites with different kinds of carbon-based fillers, such as carbon fibers, nanodiamonds, and multi-walled carbon nanotubes have attracted considerable attention [9,10,11]. As one of the most important two-dimensional carbon materials, graphene has unique mechanical properties, and its Young’s modulus and tensile strength reach 1 TPa and 130 GPa, respectively [12,13,14,15]. It has been demonstrated that adding a small amount of graphene into a polymer matrix can significantly improve the mechanical properties [16,17,18,19].

A large number of studies have shown that graphene-based fillers exhibit obvious enhancement effects on PA66. Sarac et al. [2] used the melt-blending method to prepare thermally exfoliated-GO/PA66 nanocomposites, with GO being dispersed into PA66 matrix at 300 °C and 4700 rpm in a custom-made mixer. The tensile strength and Young’s modulus of GO/PA66 were improved from 69.8 to 81.0 MPa and from 2400 to 3473 MPa, respectively. Wang et al. [20] prepared graphene sheets/PA66 nanocomposite fibers by melt-blending, and the tensile strength reached 575.6 MPa, increased by 115% compared with the pure PA66 fibers. Papadopoulou et al. [21] used a solution processing method to prepare the graphene-nanoplatelet/PA66 composite, and the tensile strength was increased from 21 to 31 MPa. The aforementioned works prepare graphene/PA66 nanocomposites mainly through physical blending, which inevitably limits the interfacial bonding between the fillers and the polymer matrix and thus restricts the enhancement on the mechanical properties of PA66 composites. Furthermore, the melt-blending method is not suitable for PA66 because the processing temperature is too high to maintain the filler dispersion state. Additionally, the solution processing method uses specific solvents with a lower dispersion state of carbon nanofillers, causing risk of damage to the filler [22].

In contrast to physical blending methods, in situ polymerization, in which the growth of polymer chains takes place in the presence of nanomaterials, is commonly utilized to prepare polymer nanocomposites with high performance. GO is one of the most important derivatives of graphene and contains abundant oxygen-containing functional groups on the surface, such as hydroxyl, epoxy, and carboxyl groups. The presence of the oxygen-containing functional groups should enable polymers to graft on the surface of GO by forming covalent bonds between the GO and polymers [23], and the GO/polymer composites have attracted attention in many application fields [24,25,26]. The rGO/PA66 nanocomposites were prepared by the in situ polymerization of rGO and PA 66 salt; however, the tensile strength was only improved from 72 to 84 MPa [27]. The limited improvement was possibly due to the agglomeration of GO. As GO carries negative net charge in aqueous solution, it will quickly agglomerate after being added into to PA66 salt aqueous solution which is an electrolyte [28,29]. Cho et al. [22] applied modified GO and carbon nanotubes (CNTs) as fillers to prepare GO/CNT/PA66 nanocomposites by the in situ polymerization method, and the tensile strength was increased from 18.2 to 40.4 MPa. This polymerization occurs at an interface between immiscible organic and aqueous phases where two monomers come into contact and undergo a polymerization reaction. However, the whole process not only involved complicated steps but also a mass of organic solvents. To the best of our knowledge, it is still challenging to develop a simple and effective method to prepare GO/PA66 nanocomposites by in situ polymerization with uniform GO dispersion.

The uniform dispersion of GO and strong interfacial bonding are the key factors in achieving high-strength GO/polymer nanocomposites [30,31,32]. In this paper, we report a facile method to achieve the better dispersion of GO in GO/PA66 salt nanocomposite by in situ precipitating adipic acid, 1,6-hexamethylene diamine and GO in ethanol solution. The GO/PA66 nanocomposite fibers are obtained by melt spinning after the in situ polymerization of the PA66 salt. In this way, the uniform dispersion of GO and the covalent interfacial bonding between GO and PA66 are achieved, which greatly enhance the mechanical properties of GO/PA66 nanocomposite fibers. The tensile strength of the fiber is improved from 265 ± 18 to 710 ± 14 MPa when the GO content is 0.3 wt%, and the Young’s modulus is increased from 1.1 ± 0.08 to 3.8 ± 0.19 GPa when the GO content is 0.5 wt%. Our work introduces a simple and practical strategy to fabricate GO/PA66 nanocomposite fibers with outstanding mechanical properties and can expand the application of PA66 in automobiles, sporting goods and other fields that require higher tensile strength. 

## 2. Materials and Methods

### 2.1. Materials

Hydrogen peroxide (30% aqueous solution, H_2_O_2_), 8000 mesh graphite (GT, purity 98%), and benzyl triethyl ammonium bromide (TEBA, purity 97%) were purchased from Shanghai Aladdin Biochemical Technology Co., Ltd. (Shanghai, China). Sulfuric acid (98% H_2_SO_4_) and potassium permanganate (KMnO_4_, purity 98%) were purchased from Shanghai Lingfeng Chemical Reagent Co., Ltd. (Shanghai, China). Adipic acid and 1,6-hexanediamine (purity 99%) were purchased from Sinopharm Chemical Reagent Co., Ltd. (Beijing, China). Anhydrous ethanol (purity 97.5%) was purchased from Wuxi Yasheng Chemical Co., Ltd. (Wuxi, China). All the chemicals and materials were not further purified.

### 2.2. Preparation of GO Aqueous Dispersion

Graphite oxide was first prepared via a modified Hummers method from graphite power [33]. An amount of 1 g of 800 mesh graphite powder was mixed with 50 mL of 98% H_2_SO_4_ at 10 °C in an ice water bath, and 4 g of KMnO_4_ was then slowly added into the mixture. The temperature of reaction system was raised to 50 °C and kept for 1 h. After cooling down to room temperature, 150 mL deionized (DI) water was added into the mixture with slow stirring until a homogeneous solution was obtained. Then, 4 mL of H_2_O_2_ was added to obtain the graphite oxide stock solution. The solution was further centrifuged at 4500 rpm and washed with DI water until the supernatant became neutral. Finally, ultrasonication was carried out for 4 h to obtain the 2 g/L aqueous GO solution. The GO solution was concentrated by using cross-flow membrane filtration to obtain the 10 g/L high-concentration GO dispersion for the convenience of subsequent solution replacement [34].

### 2.3. Preparation of GO Ethanol Dispersion by Solvent Replacement

Solvent replacement was carried out to move GO from aqueous solution to ethanol solution because PA66 salt can be formed in ethanol. Firstly, TEBA aqueous solution with a concentration of 20 g/L was prepared, and 0.5 mL of TEBA solution was slowly added as flocculant to 50 mL GO solution (10 g/L), with stirring for 15 min. Subsequently, 50 mL absolute ethanol was added into the above solution with stirring for 5 min and then centrifuged at 3000 rpm for 6 min. This step was repeated for 5 times for removing excess water and TEBA. The gelatinous GO was redispersed in ethanol with a concentration of 1 g/L and sonicated for 20 min to obtain the uniform dispersion of 1 g/L GO ethanol dispersion. 

### 2.4. Preparation of GO/PA66 Salt Nanocomposites by In Situ Precipitation

**Figure 1:**
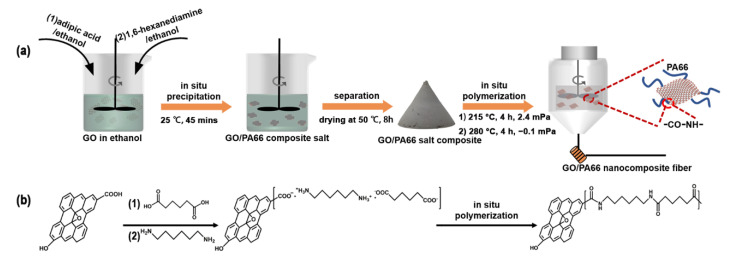
Schematic illustration of (**a**) the fabrication process of GO/PA66 nanocomposite fibers and (**b**) the reaction mechanism behind it.

The formation process of the GO/PA66 nanocomposite fibers is presented in Figure 1. The GO/PA66 salt nanocomposites with different GO contents (0, 0.1, 0.2, 0.3, 0.4, and 0.5 wt%) were prepared by an in situ precipitation method and named PA66 salt-GO-*n* (*n* is the GO content in wt%, *n* = 0, 0.1, 0.2, 0.3, 0.4, and 0.5). Taking PA66 salt-GO-0.1 as an example, first, 44.3 g of 1.6-hexanediamine was dissolved in 100 mL ethanol by stirring at room temperature, and 55.7 g of adipic acid was dissolved in 400 mL of ethanol by stirring at 40 °C. Second, 100 mL of 1 g/L of GO ethanol dispersion was added into the adipic acid ethanol solution with rapid stirring, followed by the addition of the 1.6-hexanediamine ethanol solution under rapid stirring. The in situ precipitation reaction occurred immediately, and the mixture was further stirred for 45 min. Finally, PA66 salt-GO-0.1 was obtained by filtration and drying at 50 °C for 8 h. It is noteworthy that only ethanol was used in this process, making the process green.

### 2.5. Fabrication of GO/PA66 Nanocomposite Fibers by In Situ Polymerization

GO/PA66 nanocomposite fibers were fabricated with the GO/PA66 salt nanocomposites through in situ polymerization followed by an extrusion drawing process. The GO/PA66 nanocomposite prepared with PA66 salt-GO-n is named PA66-GO-n. Taking the fabrication of PA66-GO-0.1 nanocomposite fiber as an example, PA66 salt-GO-0.1 was first dried at 50 °C for 4 h, and then 65 g DI water was added into 100 g of the nanocomposite with stirring for 5 min. The mixture was transferred to the polymerization reactor (model YT-GSH-1L, Nanjing Yantu Experimental Instrument Co., Ltd.., Nanjing, China), which was then purged with high-purity nitrogen 5 times. Subsequently, the temperature of the reaction system was raised to 215 °C and kept for 4 h. The pressure was then reduced to −0.1 MPa for removing excess water vapor, and the temperature was increased to 280 °C and kept for 4 h. Finally, the pressure was raised to 0.5 MPa with high-purity nitrogen, and the melt was directly extruded and drawn to form nanocomposite fibers.

### 2.6. Characterizations

Scanning electron microscopy (SEM, Nova NanoSEM 450, FEI Co., Ltd.., Hillsboro, OR, USA) was used to characterize the surface morphology of the GO/PA66 salt nanocomposites and GO/PA66 nanocomposite fibers with an accelerating voltage of 15 kV.

The crystal structures of GO/PA66 salt nanocomposites were characterized with X-ray diffraction (XRD, Rigaku Smart lab, Rigaku Co., Ltd.., Tokyo, Japan) using CuKα radiation (λ = 0.15406 nm), scanning the range 2θ = 10–50° with a step size of 0.02° at a 5°/min scan rate.

UV–vis absorption spectroscopy of PA66-GO salt nanocomposites prepared by different methods was conducted using an Agilent Cary 60 UV–Vis spectrometer (Agilent Co., Ltd.., Palo Alto, Santa Clara, CA, USA).

Fourier transform infrared spectroscopy (FTIR, Bruker RQUINOX55, Bruker Co., Ltd.., Karlsruhe, Germany) was used to characterize the surface functional groups of PA66, GO, and PA66-grafted GO (PA66-gGO) for the wavenumber range of 4000–600 cm^–1^ at a resolution of 4 cm^−1^. PA66-gGO was obtained as follows: the PA66-GO-0.3 nanocomposite fiber was dissolved in formic acid to remove free PA66 by using a Soxhlet extractor for 12 h. The remaining solid was washed 5 times with DI water combined with centrifugation to remove the residual formic acid, followed by a 6 h drying at 80 °C. The dried sample was named PA66-grafted GO (PA66-gGO).

X-ray photoelectron spectroscopy (XPS, Versa Probe PHI-5000, ULVAC-PHI Co., Ltd.., Kanagawa, Japan) was used to characterize the surface functional groups of PA66, GO, and PA66-gGO with a monochromatic Al Ka at 14 kV.

The thermogravimetric analyzer (TGA, NETZSCH 449 STA, NETZSCH Group, Selb, Germany) was used to study the thermal stability of the products, and the tests were carried out in a nitrogen atmosphere with the temperature ranging from 30 to 800 °C and a heat-up rate of 10 °C/min.

The melting and crystallization temperatures were determined by using differential scanning calorimetry (DSC, NETZSCH DSC 200F3, NETZSCH Group, Selb, Germany ) in a nitrogen atmosphere (0.2 MPa) with the temperature ranging from 30 to 300 °C and a heating/cooling rate of 10 °C/min.

Dynamic mechanical analysis (DMA, NETZSCH DMA 242, NETZSCH Group, Selb, Germany ) of the samples was conducted in a nitrogen atmosphere at a heating rate of 3 °C/min and a frequency of 1 Hz.

Optical microscopy (BM2000, China) was used to observe the light permeability of the nanocomposite fibers and the dispersion of GO in the fibers.

A fiber tensile tester (Jian Ke KJ-1065, Jian Ke Co., Ltd., Hefei, China) was used to measure the tensile strength and Young’s modulus of the fibers with a length of 20 mm. The tensile speed was set at 5 mm/min. The final results cited are the average and standard deviation values obtained from 10 samples.

## 3. Results and Discussion

### 3.1. Formation of GO/PA66 Salt Nanocomposites

PA66 salt is composed of adipic acid and 1,6-hexamethylene diamine connected by an ionic bond, which is the necessary precursor for PA66. In order to improve the dispersion of GO in PA66, the optimal method is to disperse GO in PA66 salt. However, it is well known that GO contains abundant oxygen-containing functional groups on its surface and exhibits a net negative charge. Once added into PA66 salt solution, which is a typical electrolyte, GO will quickly aggregate and then precipitate [28,35]. Accordingly, substituting GO into ethanol by solution replacement can reduce the charge influence effectively because the Zeta potential of GO ethanol dispersion is much lower than that of GO aqueous dispersion [29]. Therefore, this is probably to avoid GO agglomeration by using GO ethanol dispersion rather than GO aqueous dispersion.

**Figure 2:**
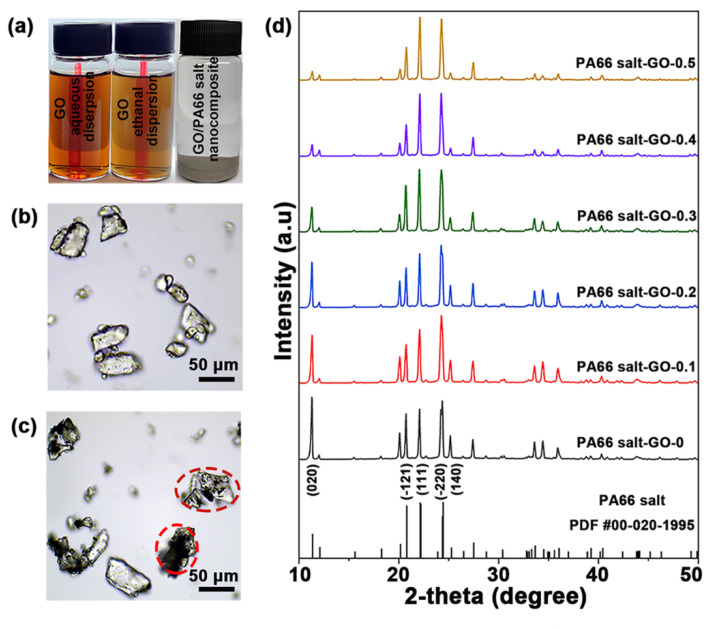
(**a**) Photos of GO aqueous dispersion (0.2 g/L), GO ethanol dispersion (0.2 g/L), and GO/PA66 salt nanocomposite formed in ethanol by in situ precipitation method. Optical micrographs of GO/PA66 salt nanocomposites formed via (**b**) in situ precipitation and (**c**) direct mixing. (**d**) XRD patterns of GO/PA66 salt nanocomposites with different GO contents.

Figure 2a shows the aqueous and ethanol dispersions of GO, and the GO/PA66 salt nanocomposite of PA66 salt-GO-0.3 in ethanol. Both the GO dispersions standing for 2 h after 5 min ultrasonication are clear and transparent, indicating that GO can maintain good dispersibility during the solution replacement. The GO/PA66 salt prepared by the in situ precipitation method is shown in Figure 2b, and all the particles are transparent, which means that GO has been uniformly dispersed in the GO/PA66 salt nanocomposite. As shown in Figure 1, the adipic acid has negative charge due to the presence of carboxyl groups, while the 1,6-hexanediamine has positive charge due to the amine groups. When the two monomers are mixed in ethanol, the acid-base neutralization will occur immediately to form PA66 salt. It is noteworthy that GO must be mixed with adipic acid first for good dispersibility. This is because if both the GO and adipic acid are negatively charged, the GO nanosheets will not be electrically attracted by adipic acid to aggregate. Figure 2c shows the GO/PA66 salt nanocomposite prepared by directly mixing the GO aqueous dispersion and the aqueous solution of adipic acid and 1,6-hexanediamine. The mixed solution was then concentrated and dried by heating to obtain the GO/PA66 salt nanocomposite. The black opaque particles in Figure 2c, highlighted in red circles, indicate the agglomeration of GO via the direct mixing method.

**Figure 3:**
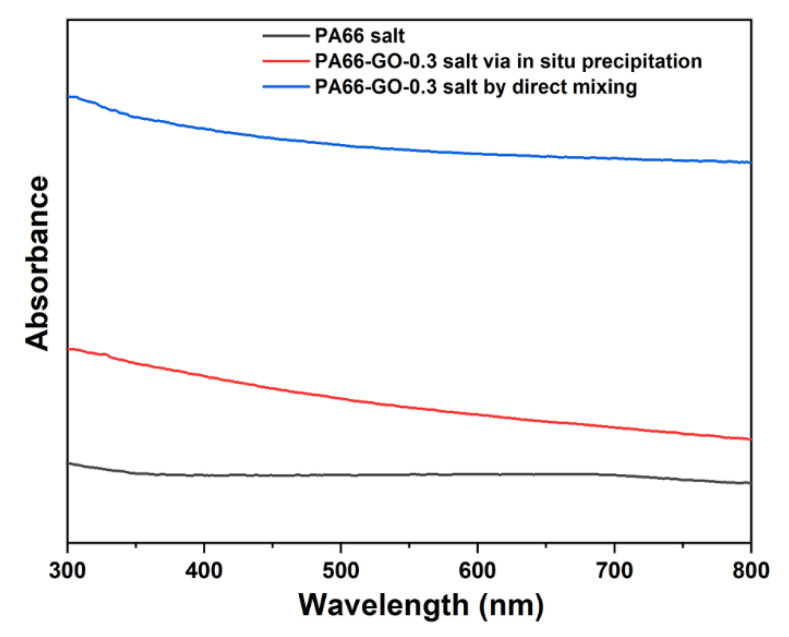
UV–vis spectra of PA66 salt, PA66-GO-0.3 salt via in situ precipitation, and PA66-GO-0.3 salt by direct mixing.

Since the aggregated GO would reduce the transmissivity of PA66 salt, the dispersity of GO in PA66 could be reflected by the absorbance of UV–vis. In order to illustrate the dispersibility of GO in PA66 salt and the advantages of the in situ precipitation, we used UV–vis spectroscopy to characterize PA66 salt, PA66-GO-0.3 salt via in situ precipitation, and PA66-GO-0.3 salt by direct mixing. From Figure 3, by the direct mixing method, the PA66-GO-0.3 salt shows much higher absorbance than PA66 salt, while the PA66-GO-0.3 salt by the in situ precipitation method has an obviously lower absorbance than PA66-GO-0.3 salt by direct mixing. The great difference in the absorbance indicates that the dispersity of GO was effectively improved by the in situ precipitation method, which is in accordance with the optical micrographs of GO/PA66 salt nanocomposites in Figure 2b,c.

**Figure 4:**
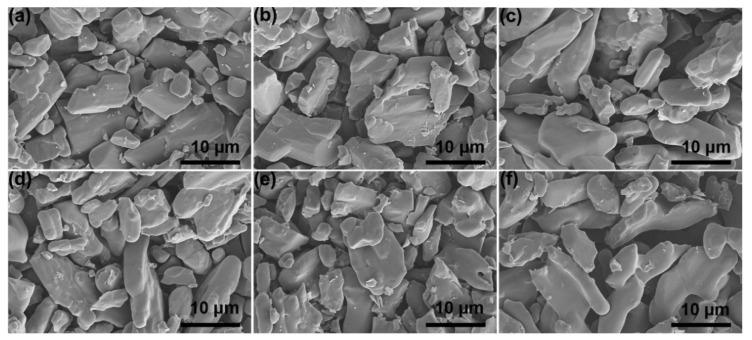
SEM images of GO/PA66 salt nanocomposites with different GO contents: (**a**) PA66 salt-GO-0, (**b**) PA66 salt-GO-0.1, (**c**) PA66 salt-GO-0.2, (**d**) PA66 salt-GO-0.3, (**e**) PA66 salt-GO-0.4, and (**f**) PA66 salt-GO-0.5.

Figure 2d shows the XRD patterns of GO/PA66 salt nanocomposites with different GO contents. The peak positions in all the XRD patterns do not show obvious changes compared with the standard PDF card of PA66 salt, confirming the formation of PA66 salt. The peak density varies with the GO content in the composites, especially the (020) face whose peak intensity is significantly weakened. This is probably due to the fact that the inorganic GO nanosheets inhibited crystallization of the PA66 salt [36]. Figure 4a–f shows the morphology of GO/PA66 salt composites with different GO contents. It can be observed that with the increase in GO content, the GO/PA66 salt has no obvious change, and no obvious GO and GO agglomerates are found in Figure 4, indicating that GO is well dispersed in PA66 salt without phase separation.

### 3.2. In Situ Polymerization of GO/PA66 Salt Nanocomposites

**Figure 5:**
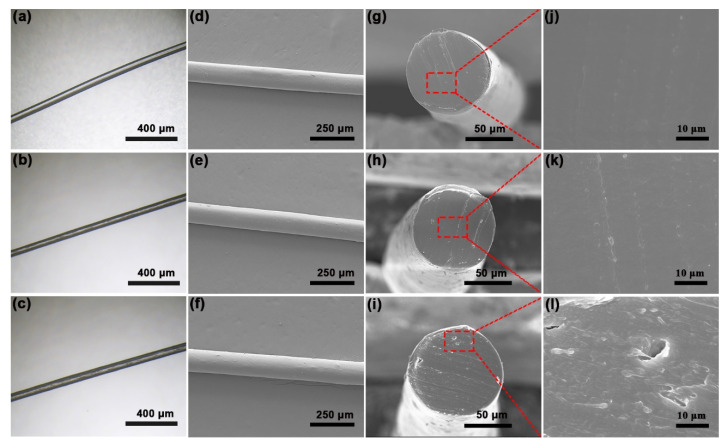
Optical micrographs and SEM images of the nanocomposite fibers: (**a**,**d**) PA66-GO-0, (**b**,**e**) PA66-GO-0.3, and (**c**,**f**) PA66-GO-0.5. Cross-section SEM images of (**g**,**j**) PA66-GO-0, (**h**,**k**) PA66-GO-0.3, and (**i**,**l**) PA66-GO-0.5.

Figure 5a–c are the optical micrographs of the GO/PA66 nanocomposite fibers with different GO contents. With the increase in GO content, the GO/PA66 nanocomposite fiber becomes dark in color, which may be caused by a partial reduction in GO. Figure S1 shows that the *I*_D_/*I*_G_ of PA66-gGO after in situ polymerization was 0.88 and *I*_D_/*I*_G_ of GO was 0.95, which reveals that the partial reduction occurred during the polymerization process [27,37]. Figure 5d–f exhibit the surfaces of GO/PA66 nanocomposite fibers; from these images, we can find that the addition of GO does not significantly change the surface morphology of the fibers. Figure 5g–l exhibit the cross-section SEM images of GO/PA66 nanocomposite fibers. From Figure 5h,k, it can be seen that there are no obvious cracks on the cross-section of the PA66-GO-0.3 fiber, which is similar to the pure PA66 fiber (Figure 5g,j). However, with the increase in GO content, as shown in Figure 5i,l, the cross-section of PA66-GO-0.5 shows slight defects. As the GO content increases, the GO nanosheets have a higher chance to interact with each other, forming bridges and leading to defects in the nanocomposites. It is noteworthy that we tried our best to examine the GO distribution via the cross-section SEM study, but no obvious GO was found. In the recent work by Yu et al. [38], the cross-section of the PET-GO fiber also showed a smooth surface. The authors ascribed this to the uniform dispersion of GO in the polymer.

**Figure 6:**
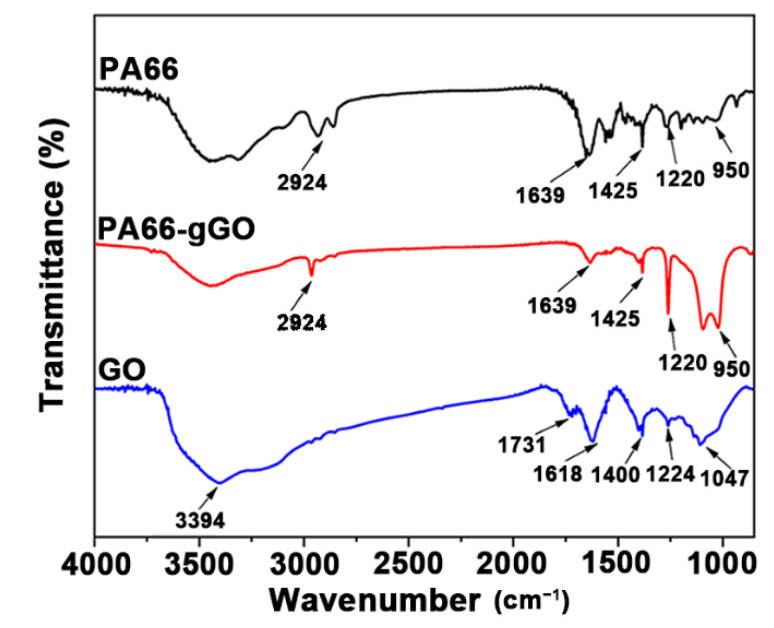
FTIR spectra of PA66, PA66-gGO, and GO.

In the in situ polymerization process of GO/PA66 nanocomposite, the amine groups of PA66 react with the carboxyl groups of GO to form new amide bonds, thus enhancing the interfacial bonding between GO and PA66 effectively. FTIR and XPS were used to directly characterize or indirectly reveal the chemical bonds between PA66 and GO. Figure 6 shows the FTIR spectra of PA66, PA66-gGO, and GO. For the GO sample, the peaks appeared at 3394, 1731, 1618, 1400, 1224, and 1047 cm^−1^ correspond to the stretching vibration of O−H, C=O from COOH groups, C=C from unoxidized graphitic domains, C−OH, C−O−C, and C−O, respectively [39]. For the PA66 and PA66-gGO samples, they both exhibit a peak at 2924 cm^−1^, which corresponds to the stretching vibration of C−H in the PA66 chain. The new peaks that appeared at 1639, 1425, 1220, and 950 cm^−1^ correspond to the stretching vibration of amide bonds (OC–NH) [40]. The existence of these new vibrations in PA66-gGO clearly indicates that PA66 was successfully grafted on GO during the in situ polymerization process by covalent bonding.

**Figure 7:**
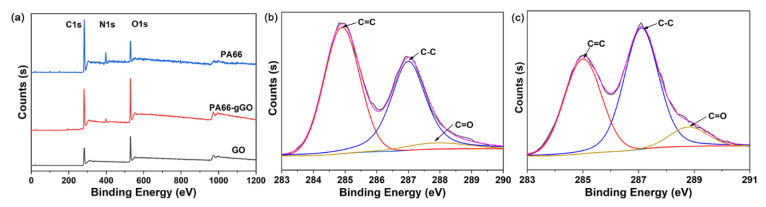
(**a**) XPS spectra of PA66 PA66-gGO and GO, (**b**) XPS peak-splitting fitting spectrogram of C 1s of GO, and (**c**) XPS peak-splitting fitting spectrogram of C 1s of PA66-gGO.

Full-scale XPS spectra of the PA66, PA66-gGO, and GO are shown in Figure 7a. All spectra contain the peaks at 285.1 eV (C 1s) and 533.0 (O 1s), but only the PA66 and PA66-gGO have a peak at 400.1 eV (N 1s). As shown in Figure 7a, the N 1s spectra of the PA66-gGO can be deconvoluted into C−N and N−H components at binding energies of 399.4 and 401.6 eV, respectively, which further confirms that PA66 was grafted onto the surface of the GO through amide bonds [41]. From Figure 7b,c, the C 1s peaks of the PA66-gGO and GO can be deconvoluted into three peaks with binding energies of 284.6, 285.5, and 289.1 eV, which are assigned to C=C, C−C, and O−C [42]. We know that the grafted PA66 via amine bond leads to an increase in the C=O content in GO which could be revealed by XPS analysis [43]. By comparing Figure 7b,c, it is clear that PA66-gGO shows an increase in C=O, which further proves the chemical bond between GO and PA66. The XPS results are consistent with the FTIR results, confirming the successful in situ polymerization of GO/PA66 nanocomposite.

### 3.3. Properties of GO/PA66 Nanocomposite Fibers

The thermodynamic parameters of the GO/PA66 nanocomposite fibers were collected by using DSC analysis (Figure S2). As shown in the second column of Table 1, the melting temperature *T_m_* shows a very small increase with the GO content. Similarly, the crystallization temperature *T_c_* also increases with the GO content. In order to compare the effects of different contents of GO on the crystallization of GO/PA66 nanocomposites, the following formula was used to calculate the crystallinity (*X_c_*):

(1) $$X_{c}\left(\%\right)=\Delta H_{c}\times 100/\Delta H_{c}^{0}$$

where $\Delta H_{c}$ is the heat of crystallization for the GO/PA66 nanocomposite and $\Delta H_{c}^{0}$ is the heat of crystallization for 100% crystalline PA66, which is 188.4 J/g [44]. The values of $\Delta H_{c}$ obtained from the DSC analysis for PA66-GO-0, PA66-GO-0.1, PA66-GO-0.2, PA66-GO-0.3, PA66-GO-0.4, and PA66-GO-0.5 nanocomposite fibers are 53.86, 48.15, 50.59, 49.62, 52.63, and 55.09 J/g, respectively. Therefore, the crystallinity of each sample was calculated to be 28.5, 25.6, 26.9, 26.3, 27.9, and 29.2%, respectively. The results of DSC experiments demonstrate that the addition of GO did not cause significant changes in the melting temperature and crystallinity of the PA66 matrix. Meanwhile, GO acted as a heterophane nucleating agent, which led to an increase in the crystallization rate of the GO/PA66 nanocomposites [45]. The crystallinity of the GO/PA66 nanocomposite fibers was also examined with XRD. During the formation of PA66 crystals, molecules are connected to each other by hydrogen bonds to form a hydrogen bond surface, and the hydrogen-bonded faces are stacked onto each other to form PA66 crystals by van der Waals force. For all the GO/PA66 nanocomposite fibers (Figure S3), two diffraction peaks appeared between 20 and 25°, which correspond to the two characteristic peaks, α1 and α2, of the α-crystalline form of PA66. α1 arose from the distance between the hydrogen-bonded chains and α2 arises from the separation of the hydrogen bond surfaces [45,46]. With the addition of GO, the characteristic peaks did not show an obvious shift, so the crystal structure of PA66 was retained in the GO/PA66 nanocomposite fibers. The DSC and XRD results revealed that GO may exert a higher effect on the amorphous region than on the crystalline regions of PA66 [47], despite the great influence of GO on PA66 salt.

**Figure 8:**
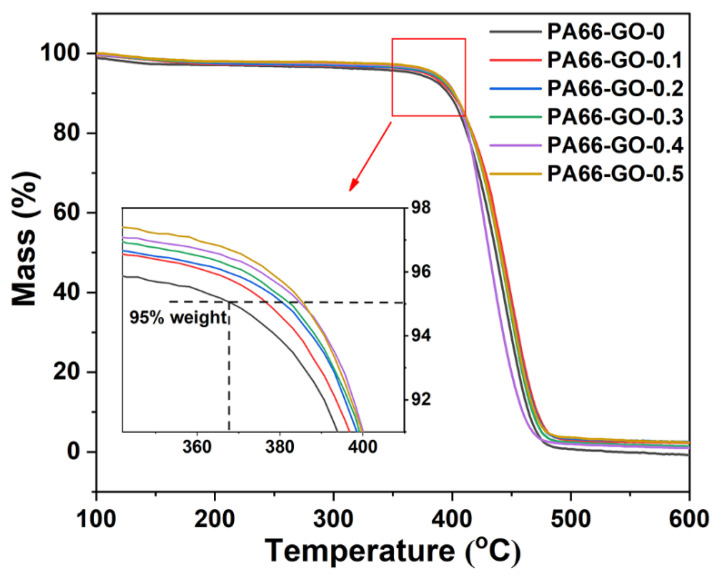
The TGA curves of PA66-GO nanocomposites with different GO contents.

Figure 8 compares the TG curves of PA66 and GO/PA66 nanocomposites with different GO contents. Overall, a rapid weight loss occurred in these samples when the temperature reached 360 °C. This was caused by the amide bond cleavage, and the PA66 molecular chain began to decompose [48,49]. Based on the enlarged TG curves (insert in Figure 7), the temperature at 5% weight loss for different samples was obtained, i.e., 368 (PA66-GO-0), 375 (PA66-GO-0.1), 380 (PA66-GO-0.2), 382 (PA66-GO-0.3), 384 (PA66-GO-0.3), and 385 °C (PA66-GO-0.5). This indicates that the thermal stability of GO/PA66 nanocomposites was improved. It should be induced by the physical barrier effect of GO, which could delay the thermal degradation of PA66 [50].

**Figure 9:**
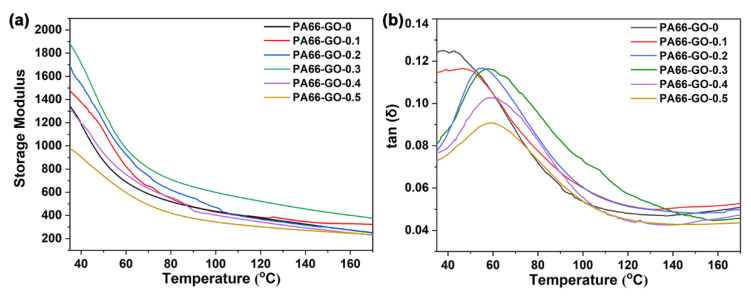
(**a**) Storage modulus (*E*’) and (**b**) tan (δ) as a function of temperature for PA66 and PA66/GO nanocomposites with different GO contents.

Figure 9 shows the storage modulus (*E’*) and loss factor (tan δ) of the GO/PA66 nanocomposites with different GO contents. The temperature corresponding to the tan δ peak can be used as the glass transition temperature (*Tg*) [3]. The DMA parameters are listed in Table 2. The *E’* of PA66-GO-*n* obviously increased at first, and decreased sharply as the GO content surpassed 0.3 wt%. The increased *E’* represents the increase in stiffness and load-bearing capacity of the composite material. The trend of *E’* is similar to the tensile strength of GO/PA66 nanocomposite fibers, and it also revealed the uniform dispersion of GO in PA66 and the strong interfacial bonding between GO and PA66. In addition, the *Tg* values of GO/PA66 composites continued to increase as the GO content increased, indicating a decrease in the free volume of GO/PA66 nanocomposites. The increased *Tg* values confirm the restricted molecular chain movement of PA66 induced by GO addition, which is in accordance with the improved thermal stability revealed by the TG study.

**Figure 10:**
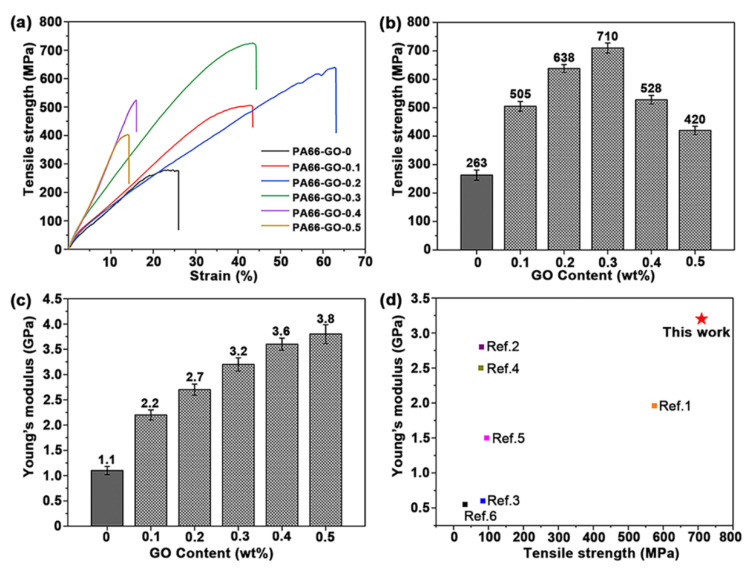
(**a**) Tensile curves, (**b**) tensile strength histogram, and (**c**) Young’s modulus histogram of GO/PA66 nanocomposite fibers. (**d**) Comparison of the tensile strength and Young’s modulus of graphene/PA66 nanocomposites (the data are shown Table S1).

Figure 10 shows the tensile strength and Young’s modulus of the PA66/GO nanocomposite fibers with different GO contents. In Figure 10a,b, the tensile strength first increases with the GO content and then decreases when the GO content is over 0.3 wt%, which indicates that the addition of GO can effectively enhance the tensile strength of the nanocomposite fibers but excess GO will agglomerate during the in situ polymerization, resulting in the formation of defects. The highest tensile strength is about 710 ± 14 MPa, which was observed from the PA66-GO-0.3 nanocomposite fiber. This tensile strength is 2.7 times that of the pure PA66 fiber (PA66-GO-0, 263 ± 18 MPa). As shown in Figure 10c, when the GO content increased to 0.3 wt% (PA66-GO-0.3), the Young’s modulus of the fiber increased to 3.2 GPa, which is nearly 190% of that of the pure PA66 fiber (P66-GO-0, 1.1 GPa). The properties of the obtained GO/PA66 nanocomposite fiber (PA66-GO-0.3) were compared with that of other reported graphene/PA66 nanocomposites. From Figure 10d, we can see that our GO/PA66 nanocomposite fiber exhibited the best tensile strength and Young’s modulus. These good properties are attributed to the uniform dispersion of GO in the GO/PA66 salt nanocomposite obtained from the in situ precipitation process and the formation of the covalent bonds between GO and PA66 via the in situ polymerization process.

## 4. Conclusions

In summary, we developed a green and scalable method to fabricate GO/PA66 nanocomposite fibers with excellent mechanical properties. The solution replacement from GO aqueous solution to GO ethanol solution was first carried out to avoid the agglomeration of GO in the PA66 salt electrolyte. The GO/PA66 salt nanocomposites with uniform dispersion of GO were then prepared by the in situ precipitation of adipic acid and 1,6-hexamethylene diamine on GO in ethanol. The GO/PA66 nanocomposite fibers were finally obtained via in situ polymerization followed by melt spinning. Compared with the pure PA66 fiber, the tensile strength of the GO/PA66 nanocomposite fiber was improved by 170% with only 0.3 wt% of GO content; the Young’s modulus was improved by 245% with 0.5 wt% of GO content. The uniform dispersion of GO in the in situ precipitation process and the chemical bonds formed between GO and PA66 during the in situ polymerization process are the main reasons for the remarkably improved strength. Our work effectively solved the problems of GO dispersion and the interfacial bonding of GO/PA66 nanocomposites. The methods in this work may provide new ideas for the preparation of high-performance PA66 composite materials by other nanofillers and provide a reference for other PA materials, such as PA56 and PA612.

## Figures and Tables

**Table 1:** DSC data of GO/PA66 nanocomposite fibers with different GO contents.

Samples	*T_m_* (°C)	*T_c_* (°C)	Δ*H_c_* (J/g)	*X_c_* (%)
PA66-GO-0	260.5	228.4	53.86	28.5
PA66-GO-0.1	261.1	228.8	48.15	25.6
PA66-GO-0.2	261.3	231.1	50.59	26.9
PA66-GO-0.3	261.8	234.0	49.62	26.3
PA66-GO-0.4	262.1	234.3	52.63	27.9
PA66-GO-0.5	262.5	234.5	55.09	29.2

**Table 2:** DMA parameters of PA66 and GO/PA66 composites.

Samples	*E*’ (MPa)	*Tan δ*	*Tg* (°C)
PA66-GO-0	1345	0.120	43.2
PA66-GO-0.1	1475	0.110	51.6
PA66-GO-0.2	1691	0.081	56.1
PA66-GO-0.3	1900	0.076	57.5
PA66-GO-0.4	1317	0.073	58.4
PA66-GO-0.5	970	0.072	59.3

## Data Availability

The data presented in this study are available on request from the corresponding author.

## Supplementary Materials

The following are available online at https://www.mdpi.com/article/10.3390/polym13111688/s1, Figure S1: Raman spectra of PA66-gGO and GO; Figure S2: DSC (a) melting and (b) crystallization curves of GO/PA66 nanocomposites; Figure S3: XRD patterns of PA66-GO-0, PA66-GO-0.1, PA66-GO-0.2, PA66-GO-0.3, PA66-GO-0.4, and PA66-GO-0.5 nanocomposite fibers; Table S1: Comparison of the mechanical properties of graphene-based PA66 composites.
