# Supplementary material for: High-Strength GO/PA66 Nanocomposite Fibers via In Situ Precipitation and Polymerization
Source: Polymers (Basel). 2021 May 22;13(11):1688. doi: 10.3390/polym13111688 (PMC8196895; doi:10.3390/polym13111688)
Supplement: Supplementary file 1 [file polymers-13-01688-s001.zip › polymers-1233603-SI.pdf]

Article

High-Strength GO/PA66 Nanocomposite Fibers via In Situ Precipitation and Polymerization

Ao Gu, Jian Wu, Liming Shen *, Xiaoyan Zhang and Ningzhong Bao *

State Key Laboratory of Materials-Oriented Chemical Engineering, College of Chemical Engineering, Nanjing Tech University, Nanjing 210009, China; 201861104102@njtech.edu.cn (A.G.); jian.wu@njtech.edu.cn (J.W.); xzhang@njtech.edu.cn (X.Z.)

* Correspondence: lshen@njtech.edu.cn (L.S.); nzhbao@njtech.edu.cn (N.B.)

Preparation of GO

1 g of 800 mesh graphite powder was mixed with 50 mL of 98% H_2SO_4 at 10 °C in an ice water bath, and 4 g of $KMnO_4$ was then slowly added into the mixture. The temperature of reaction system was raised to 50 °C and kept for 1 h. After cooling down to room temperature, 150 mL deionized (DI) water was added into the mixture with slow stirring until a homogeneous solution was obtained. Then, 4 mL of H_2O_2 was added to obtain the graphite oxide stock solution. The solution was further centrifuged at 4500 rpm and washed with DI water until the supernatant became neutral. Finally, ultrasonication was carried out for 4 h to obtain the 2 g/L aqueous GO solution. The GO solution was concentrated by using cross-flow membrane filtration to obtain the 10 g/L high-concentration GO dispersion for the convenience of subsequent solution replacement.

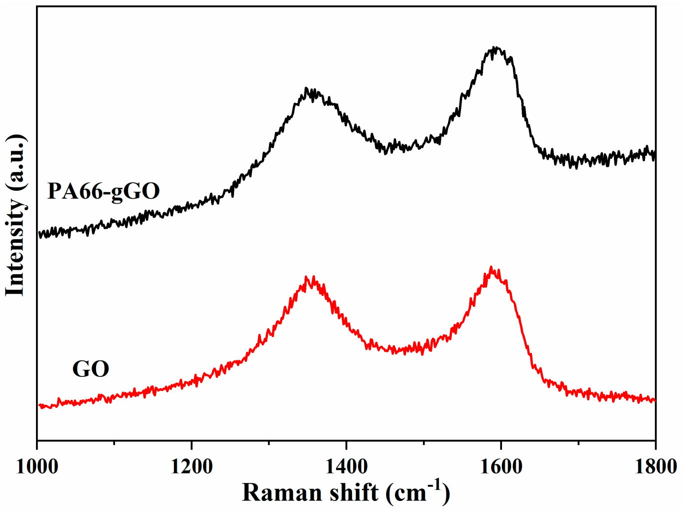

Figure S1. Raman spectra of PA66-gGO and GO.

Citation: Gu, A.; Wu, J.; Shen, L.; Zhang, X.; Bao, N. High Strength GO/PA66 Nanocomposite Fibers via In Situ Precipitation and Polymerization. *Polymers* **2021**, *13*, 1688. https://doi.org/10.3390/polym13111688

Academic Editor: Pietro Russo

Received: 8 May 2021
Accepted: 20 May 2021
Published: 22 May 2021

Publisher's Note: MDPI stays neutral with regard to jurisdictional claims in published maps and institutional affiliations.

Copyright: © 2021 by the authors. Licensee MDPI, Basel, Switzerland. This article is an open access article distributed under the terms and conditions of the Creative Commons Attribution (CC BY) license (http://creativecommons.org/licenses/by/4.0/).

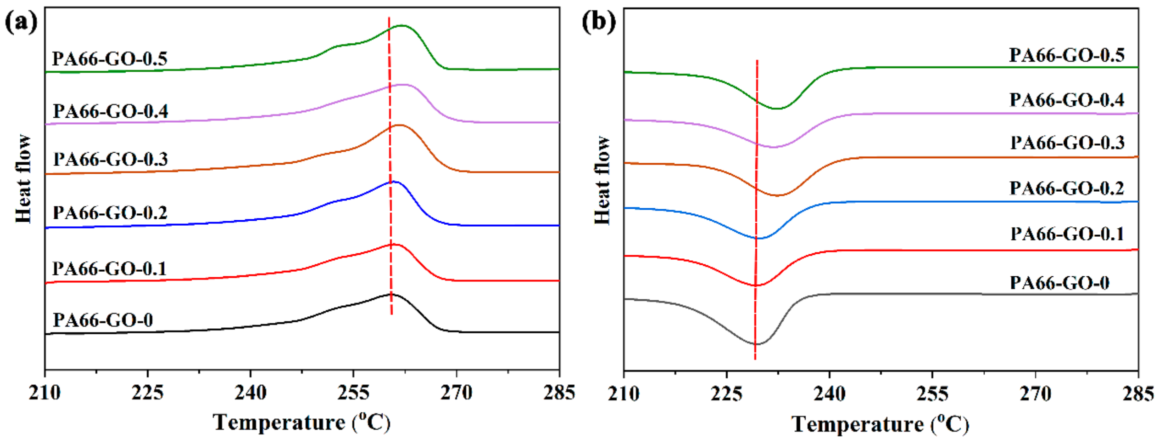

Figure S2. DSC (**a**) melting and (**b**) crystallization curves of GO/PA66 nanocomposites.

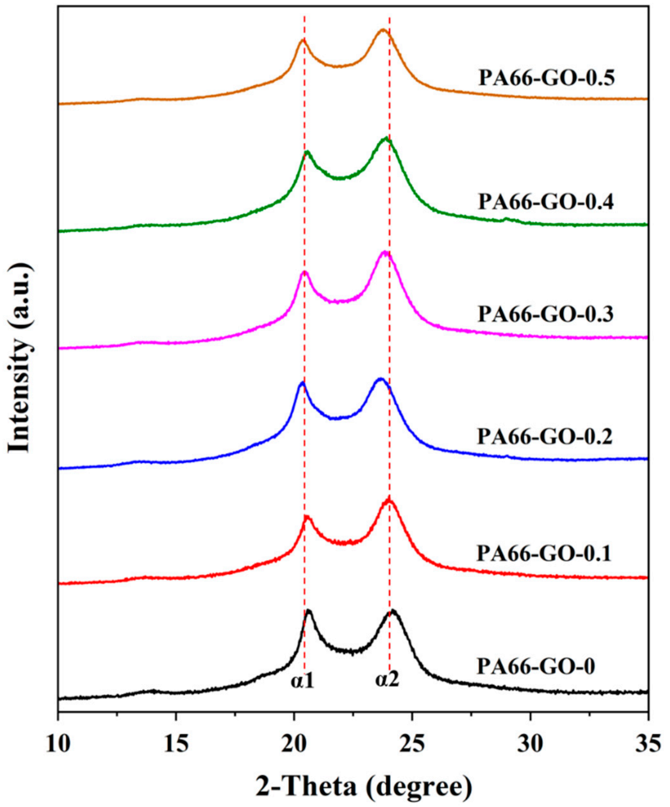

Figure S3. XRD patterns of PA66-GO-0, PA66-GO-0.1, PA66-GO-0.2, PA66-GO-0.3, PA66-GO-0.4, and PA66-GO-0.5 nanocomposite fibers.

Table S1. Comparison on the mechanical properties of graphene-based PA66 composites.

Fillers	GO content (wt%)	Tensile strength (MPa)	Young's modulus (GPa)	Sources
**GO**	0.3	710	3.2	Our work
**Graphene**	0.1	575	1.96	Ref. 1 [1]
**GNP**	1	80	2.8	Ref. 2[2]
**RGO**	1	84	0.6	Ref. 3[3]
**NC/GO**	5	78.2	2.5	Ref. 4 [4]
**RGO**	0.3	95	1.5	Ref. 5[5]
**GNP**	1	33	0.55	Ref. 6 [6]

References

1. Wang, Y. Z.; Gao, X. F.; Liu, H. H.; Zhang, J.; Zhang, X. X. Green Fabrication of Functionalized Graphene via One-Step Method and Its Reinforcement for Polyamide 66 Fibers. *Mater. Chem. Phys.* **2020**, *240*, 122288.
2. You, J.; Choi, H. H.; Cho, J.; Son, J. G.; Park, M.; Lee, S. S.; Park, J. H. Highly Thermally Conductive and Mechanically Robust Polyamide/Graphite Nanoplatelet Composites via Mechanochemical Bonding Techniques with Plasma Treatment. *Compos. Sci. Technol.* **2018**, *160*, 245–254.
3. Duan, X.; Yu, B.; Yang, T.; Wu, Y.; Yu, H.; Huang, T. In Situ Polymerization of Nylon 66/Reduced Graphene Oxide Nanocomposites. *J. Nanomater.* **2018**, *2018*.
4. Kim, J.; Oh, J.; Lee, K. Y.; Jung, I.; Park, M. Dispersion of Graphene-Based Nanocarbon Fillers in Polyamide 66 by Dry Processing and Its Effect on Mechanical Properties. Composites Part B **2017**, *114*, 445–456.
5. Wang, M.; Xin, Y.; Su, C.; Xue, F.; Xu, F.; Li, T. Morphology Evolution of a Thermotropic Liquid-Crystalline Polymer in a Polyamide 6,6 Matrix Regulated by Graphene. *J. Appl. Polym. Sci.* **2016**, *133* (31), 1–13.
6. Papadopoulou, E. L.; Pignatelli, F.; Marras, S.; Marini, L.; Davis, A.; Athanassiou, A.; Bayer, I. S. Nylon 6,6/Graphene Nanoplatelet Composite Films Obtained from a New Solvent. *RSC Adv.* **2016**, *6* (8), 6823–6831.
